# Modelling single cell dosimetry and DNA damage of targeted alpha therapy using Monte-Carlo techniques

**DOI:** 10.1007/s13246-025-01605-2

**Published:** 2025-07-28

**Authors:** Adam L. Jolly, Andrew L. Fielding

**Affiliations:** 1 School of Chemistry and Physics, Queensland University of Technology (QUT), Brisbane, Australia; 2 Centre for Biomedical Technologies, Queensland University of Technology (QUT), Brisbane, Australia

**Keywords:** Targeted alpha therapy, Monte-Carlo simulation, Radiotherapy, Nuclear medicine

## Abstract

Targeted alpha therapy (TαT) employs alpha particle-emitting radioisotopes conjugated to tumour-specific carriers to precisely irradiate tumour cells. Monte-carlo techniques have been used to accurately simulate absorbed dose and DNA damage for the four promising TαT radionuclides, Actinium-225 (^225^Ac), Radium-223, (^223^Ra), Lead-212 (^212^Pb) and Astatine-211, (^211^At). TOPAS and TOPAS-nBio, based on the Geant4 and Geant4-DNA monte-carlo codes respectively, were used to model the radioactive decay and alpha particle transport within a simplified spherical cell model. Four different sites within the cell model were used for the initial radionuclide distributions: the cell membrane layer, within the cytoplasm volume, on the nucleus surface, and within the nucleus volume. Results indicate higher absorbed doses to the nucleus per decay when radionuclides are initially located on the nucleus wall or within the nucleus volume. ^225^Ac and ^223^Ra, with longer decay chains and higher alpha yields, exhibit higher doses to the nucleus per decay compared to ^212^Pb and ^211^At. Notably, ^211^At, particularly when initially distributed within the nucleus volume or at its surface, demonstrates high relative efficacy, indicated by the absorbed dose to the nucleus per decay and number of single and double-strand breaks. These findings suggest that tumour-specific molecules should ideally target the nucleus to optimize efficacy.

## Introduction

Targeted alpha therapy (TαT) is a cancer treatment modality in which α-particle emitting radioactive isotopes are linked to tumour specific carrier molecules, for the selective internal irradiation of tumour cells [1]. To attain successful therapeutic outcomes, the choice of a suitable α-emitting radionuclide for patient specific treatment is important. Although there are many radionuclides that can be used in treatment applications, very few of them are suitable in terms of nuclear, physical, and biological properties [6, 31]. Using in-silico models can help determine appropriate radionuclides before testing on cell and murine models, ensuring that only the most appropriate α -emitting radionuclides are considered as candidates for clinical trials.

α-particles, with a range in water of 50–100 μm and a high linear energy transfer (LET) (∼80 keV/μm), are particularly suitable for treating small neoplasms or micrometastases [28, 29]. Beta (β-) emitting radioisotopes, on the other hand, exhibit longer ranges in water and lower LET (∼ 0.2 keV/μm), making them effective for treating medium to large tumours. While the extended range of β-particles is advantageous for uniformly distributing radiation dose in heterogeneous tumours, it also increases the risk of irradiating healthy tissue surrounding a tumour site. Furthermore, recent clinical studies have underscored the efficacy of TαT in overcoming observed resistance to β-particle therapy, signalling a shift in the paradigm of targeted radionuclide therapy (TRT) [26].

Of the approximately 100 alpha particle emitting radionuclides, only a few are suitable for clinical applications involving TαT. Parameters such as half-life, decay products, chemical properties, α energies and availability play a role in the selection of suitable α particle emitting radionuclides. Additionally, recent trials involving TαT have shown that the tumour uptake of the radionuclide can be imaged by using gamma (γ) radiation emitted as part of the decay chain of certain α -emitting radionuclides [13]. This makes such γ and α particle emitters more attractive.

The internalisation of radionuclides into a cell, along with the cascade of particles emitted during the decay of α-emitters, can cause DNA damage, including single-strand breaks (SSBs) and double-strand breaks (DSBs) [5, 22].

Monte-carlo codes are valuable tools for determining the energy deposition distribution within tumors following radionuclide decay [1]. Recently, significant advancements have been accomplished in the development of accurate computational mechanisms capable of simulating the transition of radiation through matter, specifically through DNA structures located within cell nuclei which is considered the primary target for ionising radiation in cells [16].

Previous studies have employed various simulation codes [9, 10], including TOPAS and TOPAS-nBio [2, 4, 14, 27, 30]. For example, Guerra Liberal et al. [14] employed TOPAS-nBio to investigate the effect cellular geometry has on self-absorbed dose and inter-cell cross-dose with α irradiation from different radionuclides using the same conditions. This highlighted TOPAS-nBio’s capability in predicting radiation dose to micrometastases and surrounding healthy cells. For their study Guerra Liberal et al. modelled the sources as discrete energy alpha sources i.e. not the full radioactive decay process. They also used Geant4’s condensed history electromagnetic physics models, noting that track structure DNA electromagnetic physics models may increase accuracy.

Berens et al. used TOPAS-nBio to count DSBs in cell nuclei from two theranostic radionuclides and showed that the TOPAS-nBio simulation of DSBs was comparable to published data for ^64^Cu [2]. El Bakkali et al. employed Geant4 to model the distribution of α-emitting radionuclides in tissue, showcasing its versatility while illustrating the intricacies of implementation [7]. Lee et al. utilised PHITS to investigate the dosimetric effects of radionuclide therapy, highlighting the code’s capabilities while also demonstrating its complexity [21]. Muraro et al. [23] outlines the challenges in a range of monte-carlo simulations as a clinical and research tool in particle therapy and concludes that expertise and data from nuclear physics experiments are important to improve the radiobiological modelling of tissue response to particle treatment.

The objective of this research is to provide a description of the dose deposition and DNA damage by some of the most promising α-emitting radionuclides currently under consideration for TαT, by means of accurate monte-carlo track structure simulations. This work primarily focuses on utilising the capabilities of the monte-carlo track structure code TOPAS-nBio to accurately model the complete transport of α particles and other associated decay products of four alpha emitting radionuclide candidates for clinical TαT within microbiological systems. The focus is on how and where physical dose is absorbed. The work does not attempt to take account of temporal effects that influence internal radionuclide therapy including for example uptake, DNA damage repair, and the reduction in activity due to the radioactive decay process.

## Methods

### Monte-Carlo simulations

The TOPAS [22] (TOol for PArticle Simulations) monte-carlo code (version 3.9) [27, 30], and extension TOPAS-nBio [30], were used to simulate the effects of targeted α emitting therapy (TαT) at the micro-and nano-scale in a simple cell geometry model. TOPAS and TOPAS-nbio are codes that wrap and extend the Geant4 (GEometry ANd Tracking) and Geant4-DNA simulation toolkits [15, 20]. The TOPAS and TOPAS-nBio codes offer the user a simpler interface combined with the validated physics modelling capabilities of the Geant4 codes.

### Cell geometry and radionuclide distribution

A simple cell model consisted of two homogeneous concentric spheres representing the cell and its nucleus. The cell geometry has a radius of 10 μm, while the nucleus geometry has a radius of 5 μm. The cell is centred in a water cube, side lengths of 30 μm. This model follows the work of a number of authors, enabling comparison with established dose, S-values and DNA strand break counts [4, 14, 17]. Simulations all used the material G4_WATER within the cell and nucleus geometry. G4_WATER is included in the TOPAS materials database and has a density of 1 g/cm^3^.

To evaluate the potential effect that the location of the initial radionuclide distribution in the cell geometry has on absorbed dose to the target nucleus, 100 α emitting radionuclides per cell were randomly distributed in four sub-volumes (a distributed source model in TOPAS):

- A 0.01 μm thin layer on the outer cell wall,
- Within the cytoplasm volume,
- A 0.01 μm thin layer on the nucleus wall and
- Within the nucleus volume.

The number of radionuclide sources (100) was chosen to balance calculation time with uncertainties on averaged scoring quantities.

### Physics models

Simulations utilised an extended physics model list for the cell geometry *outside of the nucleus* which included g4em-standard_opt0, g4h-phy_QGSP_BIC_AllHP, g4decay, g4ion-binarycascade, g4h-elasticHP, g4stopping, and g4radioactivedecay. *In the nucleus*, the electromagnetic physics of photons and electrons was modelled either using g4em-standard_opt0 or g4em-dna. Unless stated otherwise default TOPAS parameters were used throughout this work.

### Radionuclide decay chains

Α emitting radionuclides are gaining prominence in cancer therapy due to their unique properties. The radionuclides investigated in this research are outlined below in Table 1 including a brief outline of clinically relevant properties.

**Table 1:** Clinically relevant radionuclides used for TαT and their properties

Radionuclide	Half-line	Targeting potential	Therapeutic applications	Chemical properties	Challenges
^225^**Ac**	~ 9.92 d	Conjugates with: Antibodies Peptides Molecules Nps^a^	Leukaemia mCRPC^b^ Breast cancer Brain cancer	Actinide Binds with: Chelators NPs^a^	Production Purification Handling
^223^**Ra**	~ 11.4 d	Exhibits preferential uptake in: bone tissue	mCRPC^b^ Bone metastases	Alkaline metal Administrated as: RaCl^c^ RaCl_2_^4^ Binds to bone tissue	Hematologic toxicity Handling
^212^**Pb**	~ 10.6 h	Conjugates with PSMA ligand^e^	mCRPC^b^ NETs^f^	Heavy metal Binds with: Chelators	Production: Recent generator advancements [26]
^211^**At**	~ 7.2 h	Conjugates with: Antibodies Peptides Molecules Lipsomes	Brain tumours Thyroid cancer	Halogen Similar to Iodine Iodine based chemical binding	Scarcity Complex chemistry

^a^Nano particles, ^b^metastatic castration resistant prostate cancer, ^c^Radium Chloride, ^d^Radium Dichloride, ^e^Prostate specific membrane antigen, ^f^Neuroendocrine tumour

For each simulated primary parent radionuclide, the complete decay chain to stability was generated and transported by the Geant4 physics models mentioned in Sect. “Monte-Carlo simulations”. Full decay schemes can be found in Appendix 1 where α emitting progeny are outlined in red. Primary radionuclides, βemitting progeny and final stable states are outlined in black.

In order to further understand the effect of modelling the full radioactive decay and for comparison with the work of others simulations were also performed using isotropic alpha only sources. Alpha energies were modelled using a discrete spectra with energies equal to those emitted by the parent and daughters in the decay chains, see Appendix 1. Relative weights of the discrete energies were also set to match those in the decay chains.

### Scoring quantities

#### Absorbed dose

The TOPAS DoseToMedium scorer measures the energy absorbed within a defined volume from both the primary radionuclides and the particles they produce as they move through the volume The absorbed dose to medium was calculated for all four source distribution-target geometry configurations described previously. The simulations were performed for each of the four primary radionuclide sources listed in Table 1. Two scorers were used, the first scored the dose to the nucleus from all particles while a second scorer used a filter to score the dose to the nucleus due to alpha particles. Each simulation was repeated 20 times and the mean and standard deviation of the absorbed dose to the nucleus calculated over the 20 statistically independent simulations. The mean absorbed doses from both scorers were converted to absorbed doses per primary decay. It is worth noting the mean absorbed dose per decay to a target location such as the nucleus due to activity accumulated in a particular region (e.g. cell wall, cytoplasm or nucleus) is sometimes referred to as the *S-value* in the literature.

#### Number of particles crossing the nucleus surface (hits)

The number of particles crossing the nucleus OuterCurvedSurface was also scored using the SurfaceTrackCount surface scorer of the TOPAS code. Again, two scorers were used, one for all particles and one that filtered for alpha-particles only.

#### Quantifying DNA damage with DBSCAN

Density-Based Spatial Clustering of Applications with Noise (DBSCAN) is an algorithm that can be used to estimate potential DNA damage from the spatial clustering of ionisation events in water [8]. The spatial distribution of ionisations by charged particles are processed using a deposited energy dependent probability function. Interactions within a cell are modeled as having a probability of causing DNA damage that is assumed to increase linearly with energy and then remain constant, ranging from 0 for energies below a threshold of 5 eV to 1 for energies of 37.5 eV or greater. A subsequent filtering process assumes that the molecular DNA occupies 16% of the nucleus volume therefore a random sampling of 16% of the ionisation events in the nucleus is used to model the probability that an ionisation event will occur through direct interactions on a DNA strand and scores it as a single strand break (SSB). The DBSCAN algorithm then searches for SSB ionisation events separated by less than 3.2 nm which is approximately the length of 10 base pairs. These are scored as double strand breaks (DSBs) which are then further classified as simple (sDSB) or complex (cDSB) double strand breaks. The distinction between these two types of DSB can be defined as follows, sDSBs involve the breaking of both DNA strands at a single site whereas cDSBs involve the simultaneous occurrence of multiple breaks or lesions within the aforementioned distance limit (3.2 nm) on the DNA molecule [11]. While both sDSBs and cDSBs involve double strand DNA breaks, cDSBs are characterised by the presence of proximal high density clustered breaks often resulting in more complex forms of DNA damage such that repair of cDSBs poses greater challenges and carries a higher risk of genomic instability compared to sDSBs. Density clustered breaks often result in more complex forms of DNA damage such that repair of cDSBs poses greater challenges and carries a higher risk of genomic instability compared to sDSBs.

The TOPAS implementation of the DBSCAN scorer was used to produce information on the ionisation clusters including for each particle track that crosses the nucleus volume, the number of single strand breaks, simple double strand breaks, complex strand breaks, cluster sizes and cluster size weights. The simulations were performed for each of the four radionuclide sources and initial source locations. The track structure g4em-dna physics models need to be used in the nucleus for these simulations. Each simulation was also repeated 20 times and the mean and standard deviation of the SSB, sDSB, and cDSB quantities averaged over the 20 statistically independent simulations. The average SSB, sDSB, and cDSB per parent decay were then calculated.

#### Particle energy spectra

A further computational experiment was performed to investigate the energy spectra of the particles generated in the simulations. Simulations were performed that placed 1 × 10^4^ radionuclides at the centre of the 10 μm spherical cell model filled with the material G4_WATER. Phase space files were generated of particles crossing spherical surfaces with radii of 1 nm and 1 μm from the centre of the sphere. This was performed using the g4em-dna electromagnetic physics.

## Results

### Absorbed dose to nucleus

**Fig. 1:**
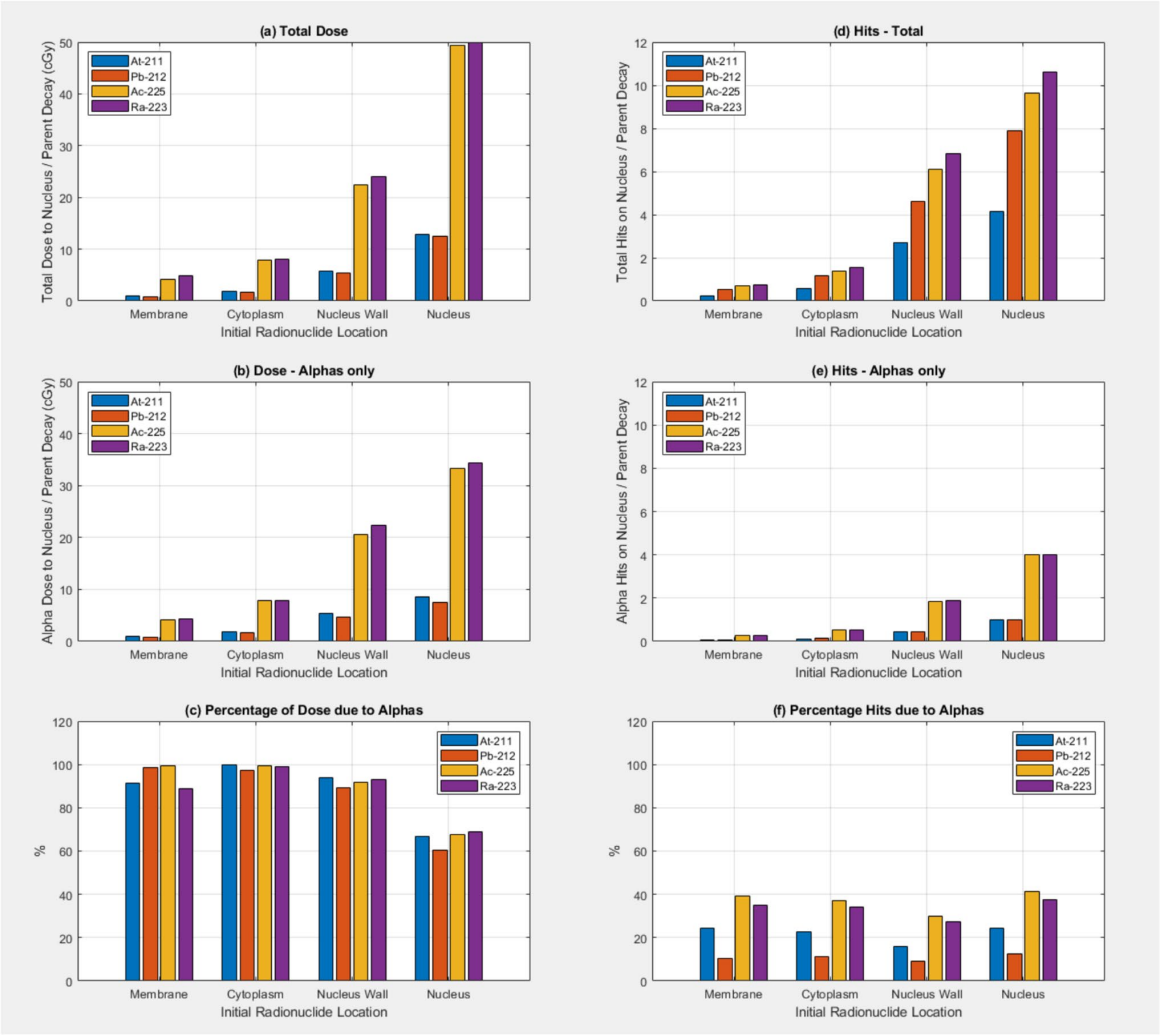
Absorbed dose to the nucleus (in cGy) per parent radionuclide decay as a function of the original source location for **a** All particles, **b** Alpha particles only, **c** percentage of the dose due to Alpha’s. The number of particles/parent decay crossing the nucleus outer surface (Hits) is shown for (**a**) All particles, (**b**) Alpha particles only, **c** percentage of the hits due to Alpha’s. Results are shown for the four alpha emitting radionuclides At-211, Pb-212, Ac-225, and Ra-223. Full radionuclide decay source models were used and g4em-standard_opt0 physics was used throughout the cell model including the nucleus.

Absorbed dose to the nucleus per decay was scored for the four different initial radionuclide source locations within the cell model. Results shown in Fig. 1a–c indicate that the highest absorbed dose to the nucleus/decay is observed when the radionuclides are located within the nucleus itself. Absorbed doses to the nucleus/decay are higher for the ^225^Ac and ^223^Ra radionuclides compared to ^211^At and ^212^Pb radionuclide sources. Also shown in Fig. 1 is the contribution of the absorbed dose to the nucleus directly from alpha particles. Figure 1d–f shows the number of particles crossing the nucleus (Hits) for all particles and the alpha particle contribution. Simulations for data in Fig. 1 used the full radionuclide decay models and g4em-standard_opt0 physics throughout the cell model including the nucleus.

**Fig. 2:**
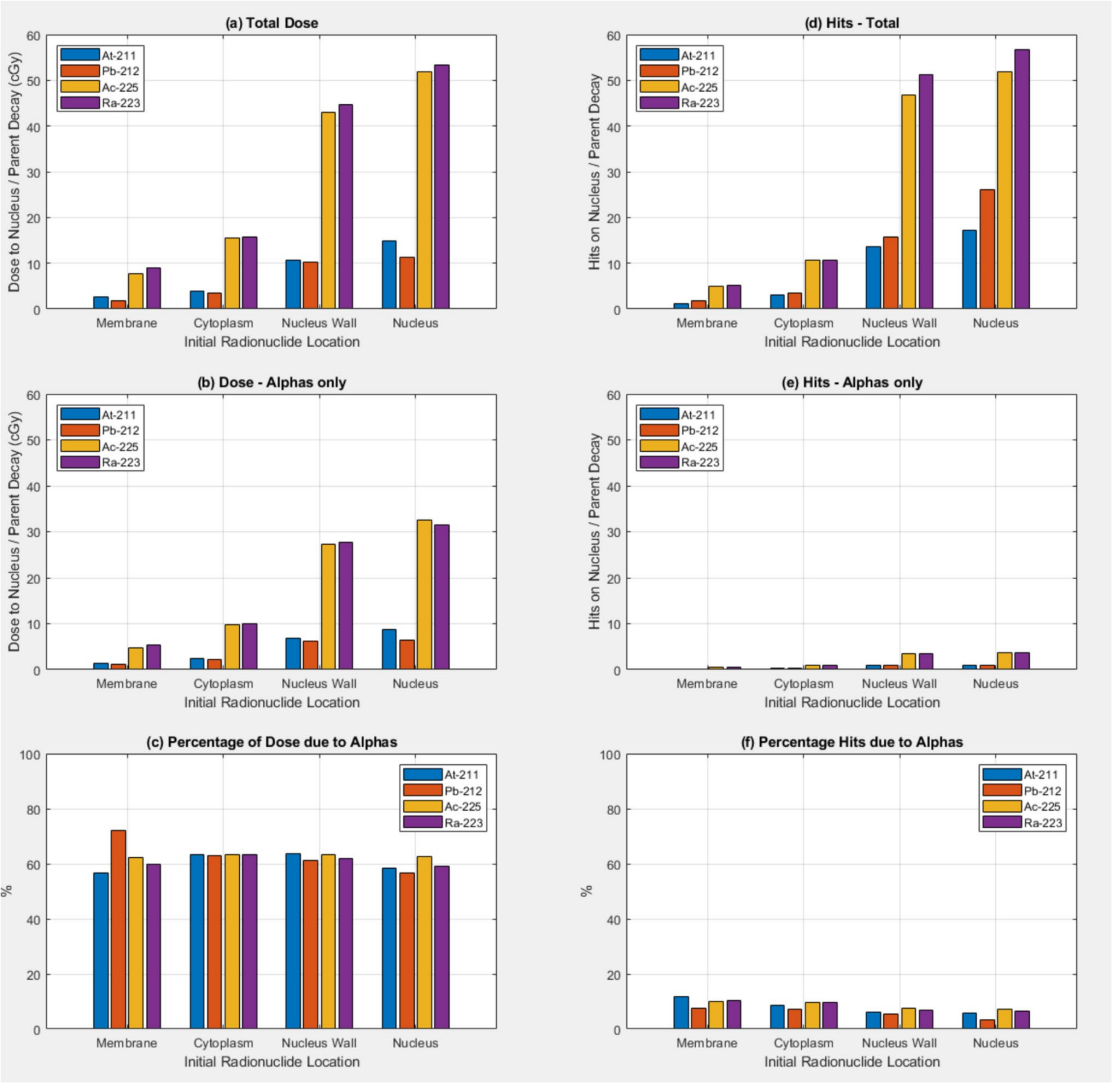
Absorbed dose to the nucleus (in cGy) per parent radionuclide decay as a function of the original source location for **a** All particles, **b** Alpha particles only, **c** percentage of the dose due to Alpha’s. The number of particles/parent decay crossing the nucleus outer surface (Hits) is shown for **a** All particles, **b** Alpha particles only, **c** percentage of the hits due to Alpha’s. Results are shown for the four alpha emitting radionuclides At-211, Pb-212, Ac-225, and Ra-223. Full radionuclide decay source models were used and g4em-dna physics was used in the nucleus.

Figure 2 shows absorbed dose to the nucleus/decay and Hits to the nucleus surface/decay when full radionuclide decay source models were used and g4em-dna physics models were used in the nucleus. Increase in the absorbed dose to the nucleus/decay and Hits to the nucleus surface/decay are not as large when the sources are in the nucleus compared to the data in Fig. 1 with the g4em-standard_opt0 physics used throughout the cell including the nucleus. The contribution of the alpha’s to both the dose to the nucleus/decay and Hits to the nucleus surface/decay doesn’t show the same dependance on the initial location of the sources in contrast to the data in Fig. 1. The contribution of the absorbed dose to the nucleus due to the alpha’s is reasonably independent of the initial source location at around 60%.

**Fig. 3:**
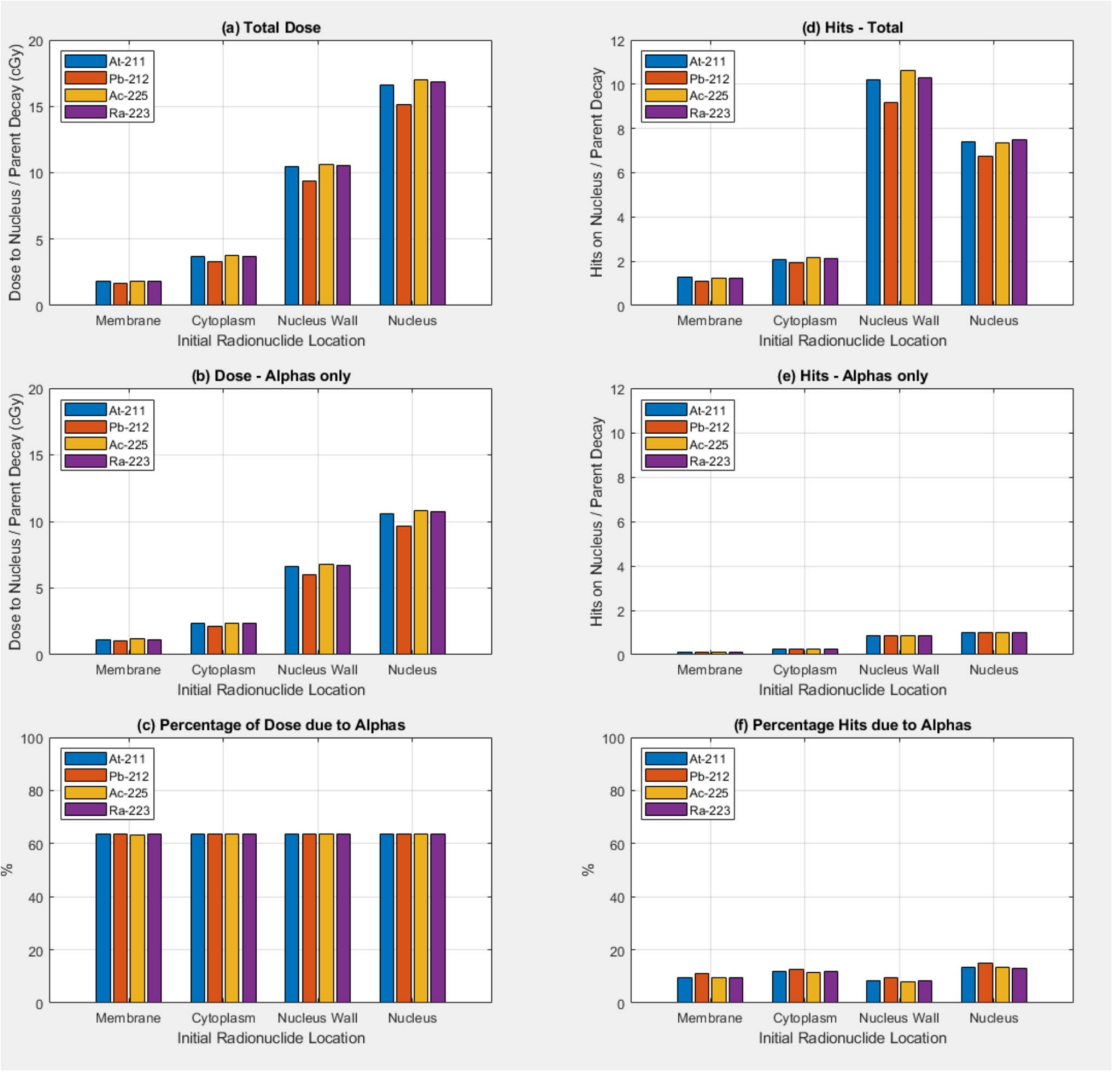
Absorbed dose to the nucleus (in cGy) per parent radionuclide decay as a function of the original source location for **a** All particles, **b** Alpha particles only, **c** percentage of the dose due to Alpha’s. The number of particles/parent decay crossing the nucleus outer surface (Hits) is shown for **a** All particles, **b** Alpha particles only, **c** percentage of the hits due to Alpha’s. Results are shown for the four alpha emitting radionuclides At-211, Pb-212, Ac-225, and Ra-223. Isotropic discrete energy alpha sources were used and g4em-dna physics was used in the nucleus.

Figure 3 shows absorbed dose to the nucleus/decay and Hits to the nucleus surface/decay when discrete energy alpha source models were used and g4em-dna physics models were used in the nucleus. The dose to the nucleus/decay shows a similar trend to the data in Fig. 1 as a function of the location of the sources. However with the discrete energy alpha sources there is no longer the large differences between the radionuclides. The number of hits to the nucleus surface/decay also doesn’t show the variation between radionuclides. There is however a reduction in the number of Hits to the nucleus surface/decay when the sources are located in the nucleus compared to when the sources are on the nucleus surface. With this discrete energy alpha source model on average only the single alpha particle/decay crosses the nucleus surface, which is not surprising as only one alpha is emitted per decay. With the full decay models used for the data of Figs. 2 and 3 multiple alpha’s are emitted/decay due to the daughter decays also being modelled.

Table 2 lists the calculated dose values of ^211^At for different source and physics models compared with published dose values from research conducted by Guerra Liberal et al. [14].

**Table 2:** Comparison of absorbed dose to the nucleus/decay for ^211^At from different source locations: on the cell membrane, in the cell cytoplasm, and in the nucleus

Study	Source model	Total/alpha dose	EM physics in nucleus	Source location		
				Mem	Cyto	Nuc
Current work—total	Full decay	Total	g4em-dna	2.59 (1.47)	3.85 (1.31)	14.92 (1.42)
Current work—alpha	Full decay	Alpha	g4em-dna	1.47 (0.55)	2.44 (0.83)	8.71 (0.93)
Current work—total	Full decay	Total	g4em-standard_opt0	0.93 (0.56)	1.79 (0.42)	12.88 (0.50)
Current work—alpha	Full decay	Alpha	g4em-standard_opt0	0.93 (0.56)	1.79 (0.42)	8.61 (0.50)
Current work—total	Alpha	Total	g4em-dna	1.81 (0.58)	3.67 (0.94)	16.63 (1.08)
Current work—alpha	Alpha	Alpha	g4em-dna	1.15 (0.37)	2.34 (0.60)	10.58 (0.67)
Guerra Liberal et al.—alpha	Alpha only	Total	g4em-standard_opt4	1.04	1.98	8.26

Results are shown for the dose to the nucleus/decay due to all particles, only alpha particles with full decay models and for alpha only sources with discrete energies. Uncertainties in parentheses are standard deviations from the mean of the 20 independent simulations. Results for different EM physics lists in the nucleus are shown to enable comparison with [14]

### Quantifying DNA damage with DBSCAN

**Fig. 4:**
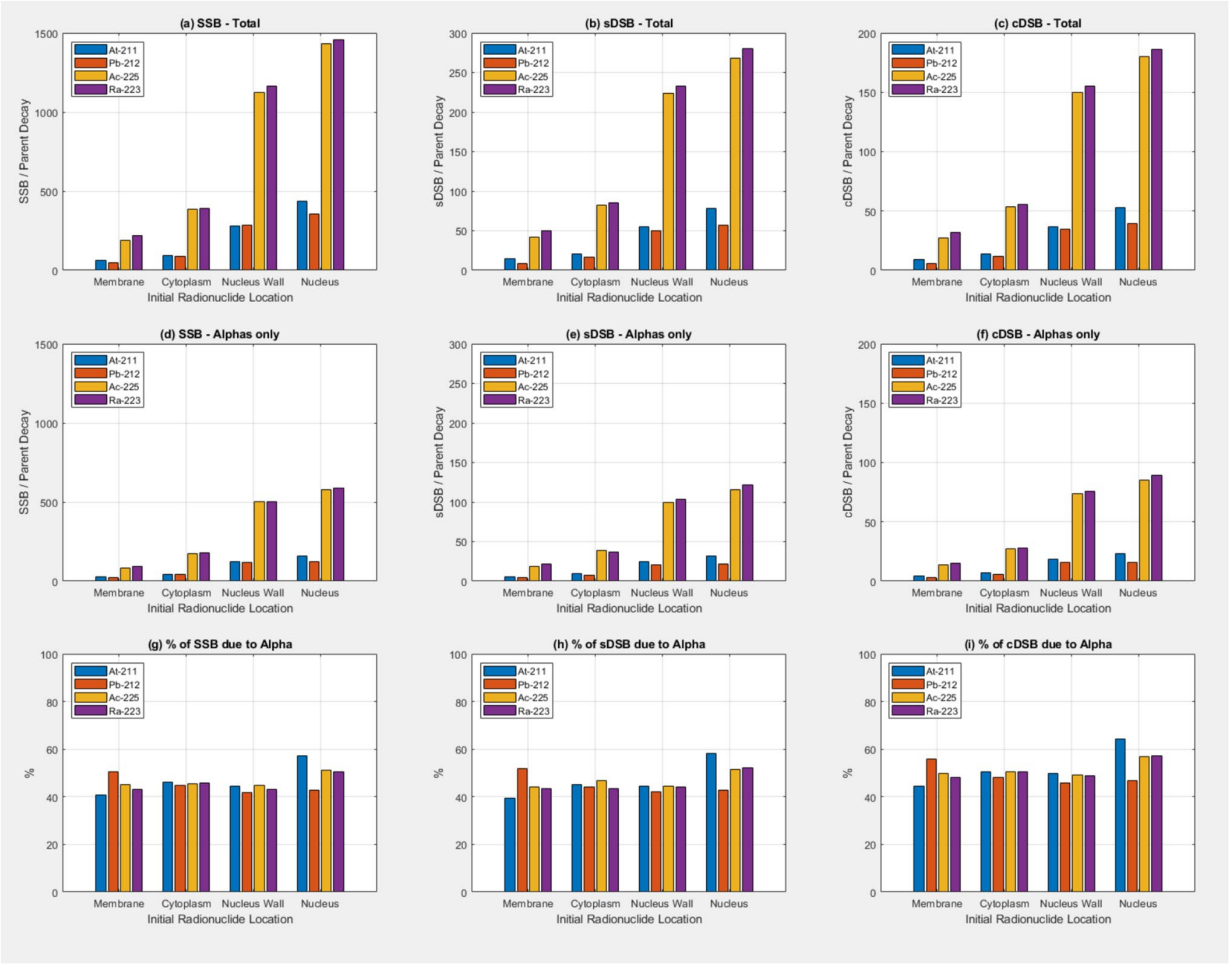
**a**–**c** Number of Single Strand Breaks (SSB), simple Double Strand Breaks (sDSB), and complex Double Strand Breaks (cDSB) as a function of radionuclide and initial radionuclide location due to all particles. **d**–**f** SSB, sDSB, and cDSB as a function of radionuclide and source location due to alpha particles only. **g**–**i** Percentage of SSB, sDSB, and cDSB as a function of radionuclide and source location due to alpha particles. Full radionuclide decay models are used and g4em-dna physics in the nucleus

Figure 4 shows the quantity of SSBs, sDSBs and cDSBs generated by 100 randomly distributed radionuclides initially located in each of the four sub-volume compartments of the cell model using the DBSCAN algorithm. SSBs, sDSBs, and cDSBs all increase with initial radionuclide source proximity to the nucleus, with the high yield α emitters (^225^Ac and ^223^Ra) displaying a higher yield with the radionuclide sources initially closer to the nucleus. As for the absorbed dose to the nucleus data in Fig. 2a–c the increase in yield of strand breaks is maybe not as high as one might expect when the sources are initially located in the nucleus. Around 40–50% of the strand breaks are due to the alpha particles directly. ^211^At shows a slightly higher yield compared to ^212^Pb when sources are located in the nucleus.

**Fig. 5:**
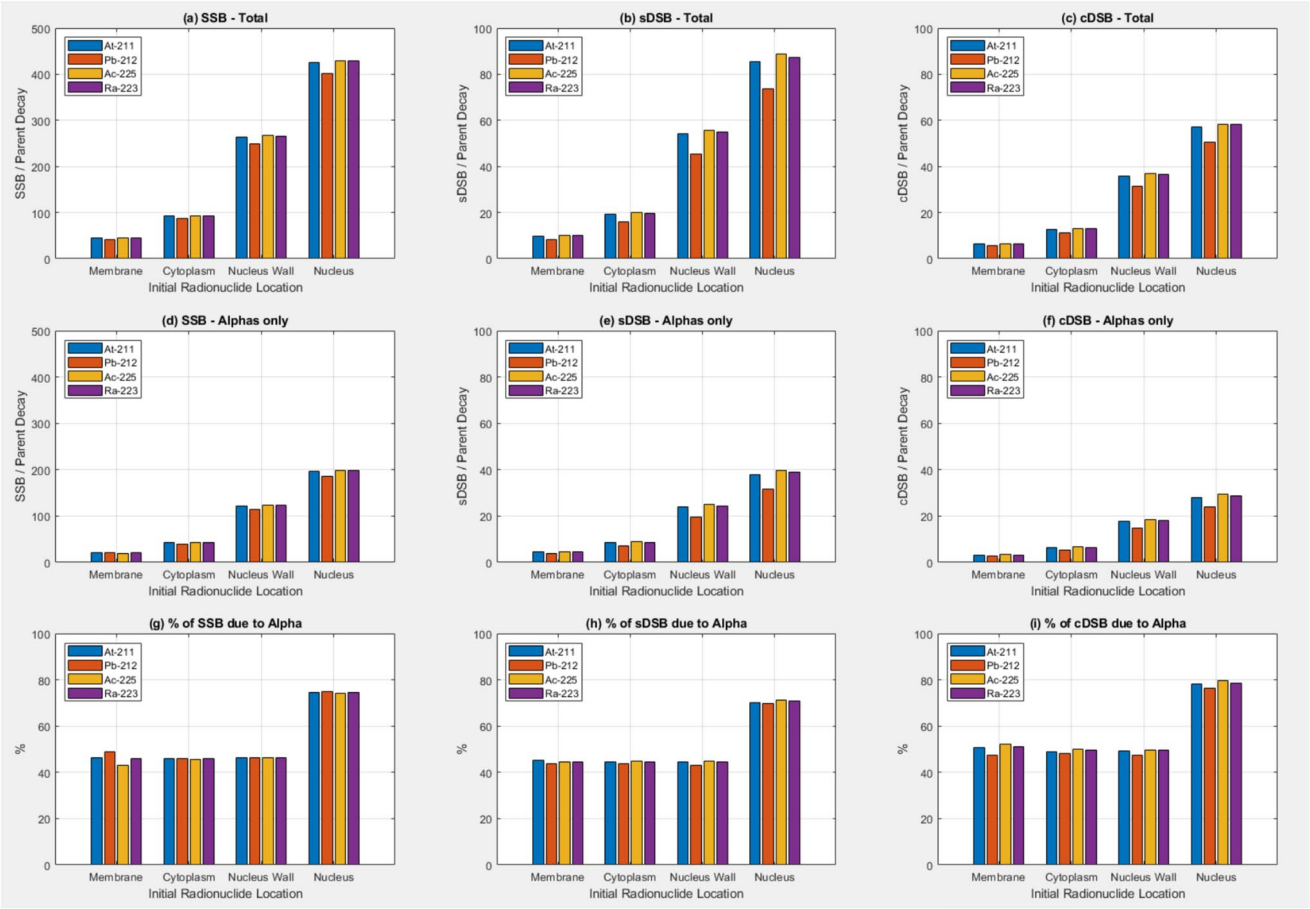
**a**–**c** Number of Single Strand Breaks (SSB), simple Double Strand Breaks (sDSB), and complex Double Strand Breaks (cDSB) as a function of radionuclide and initial radionuclide location due to all particles. **d**–**f** SSB, sDSB, and cDSB as a function of radionuclide and source location due to alpha particles only. **g**–**i** Percentage of SSB, sDSB, and cDSB as a function of radionuclide and source location due to alpha particles. Isotropic discrete energy alpha sources were used and g4em-dna physics was used in the nucleus.

**Fig. 6:**
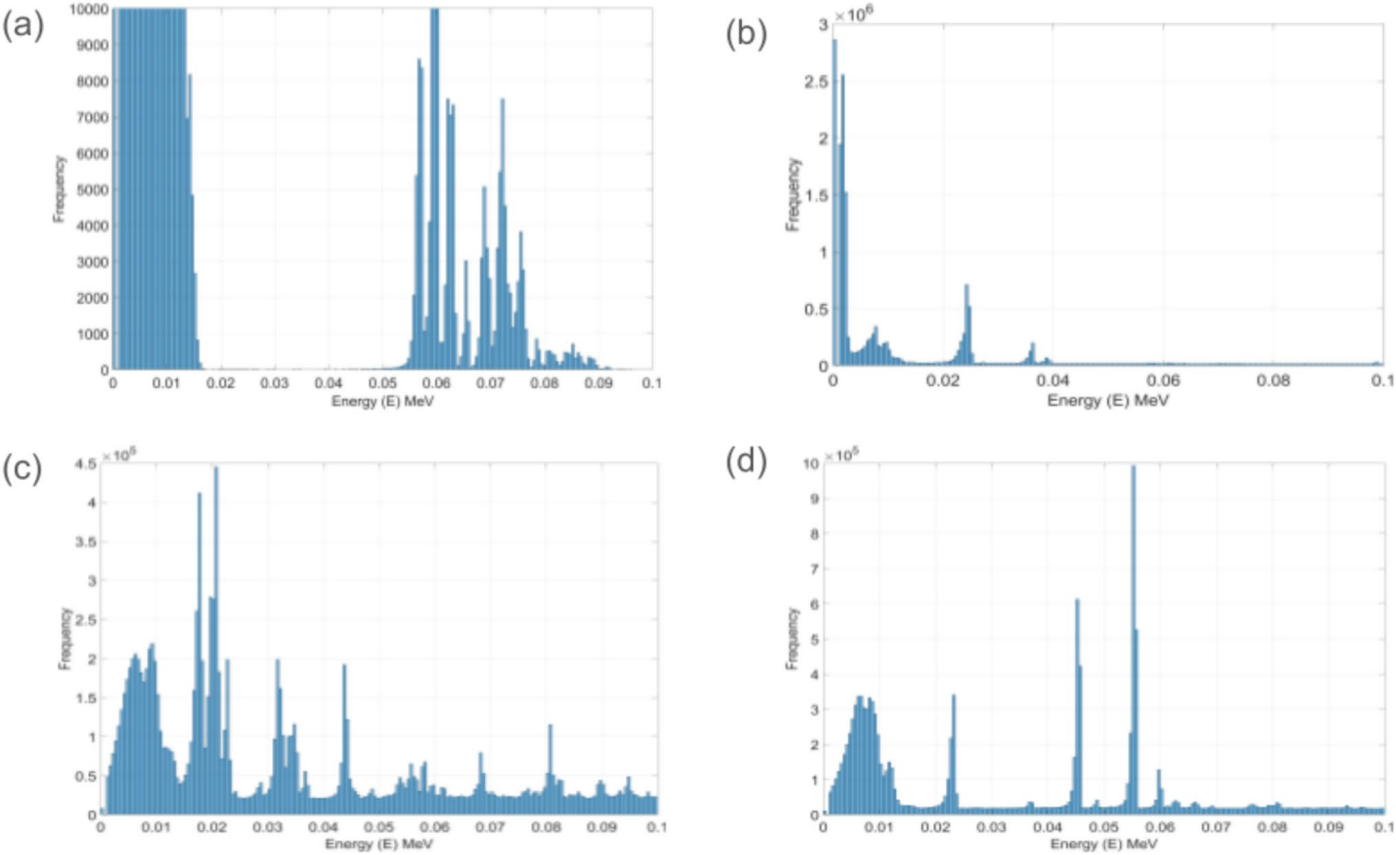
Electron energy spectra for the different radionuclide decay sources **a**^211^At, **b**^212^Pb, **c**^225^Ac, and **d**^223^Ra. Phase spaces were scored on a spherical surface 1 μm from the sources initial location at the centre of the sphere. g4em-standard_opt0 was used to model electromagnetic physics

Figure 5 shows similar data to Fig. 4 for the situation where the sources are modelled as isotropic discrete energy alpha sources. A larger increase in the yield of strand breaks is observed when the sources are located in the nucleus, relative to sources being initially located outside the nucleus for these source models compared to the full radioactive decay models. The variation in strand break yields for the different radionuclides is not observed in the data in Fig. 6. The percentage of strand breaks due to alpha particles directly is higher when the sources are initially in the nucleus.

### Particle energy spectra

Figure 6 shows the result of simulations that placed 1 × 10^7^ radionuclides at the centre of a 10 μm sphere and scored phase space files for particles crossing a spherical surface of radius 1 μm from the centre of the sphere. Full radioactive decay was modelled and the g4em-standard_opt0 condensed history electromagnetic physics used.

## Discussion

Figure 1 shows the dependence of the absorbed dose to the nucleus per decay as a function of the initial location of the radionuclide sources. The dose to the nucleus increases as the location of the sources gets nearer to the nucleus. It is clearly important to get the alpha-emitting radionuclides into the nucleus of the tumour cells to maximise absorbed dose. Two of the alpha-emitting radionuclides show significantly higher absorbed doses to the nucleus/decay, the ^225^Ac and ^223^Ra than the other two, ^212^Pb and ^211^At. The more complex decay chains (shown in Appendix 1) for ^225^Ac and ^223^Ra explains this effect with multiple alpha’s being emitted/primary decay of the parent. Figure 1b shows this effect is due to the direct dose deposition of the alpha-particles that follow a similar trend with initial source location. Figure 1c shows that when the sources are initially located outside the nucleus most of the dose to the nucleus/decay is due to the alpha’s, around 90–100%. When the sources are initially located in the nucleus then the relative contribution of the dose to the nucleus is lower, around 60–70% of the total dose to the nucleus. The extra dose is being contributed by the daughter nuclei, with its small recoil energy and also the short range low energy electrons from the alpha particle ionisations as well as auger electrons and internal conversion electrons with discrete energies. Auger electrons are known for being densely ionising, causing significant DNA damage, and are increasingly used in their own right as targeted radionuclide therapy agents [18]. Figure 1d–f shows the number of particles crossing the nucleus wall. The total number of particles crossing into the nucleus increases as the initial location of the sources moves closer to the nucleus. Figure 1e shows the number of alpha particles crossing the nucleus wall, and essentially reflects the number of alpha particles/primary decay reaching the nucleus. When the initial source location is outside the nucleus not all the alpha particles enter the nucleus. It is also worth noting these are averages over 20 statistically independent simulations and that the condensed history technique was used to model the electromagnetic physics through use of the g4em-standard_opt0 physics list in the whole cell geometry.

Figure 2 shows results for similar simulations as shown in Fig. 1 except in this case the g4em-dna physics was used in the nucleus. Doses to the nucleus/decay when the initial location of the sources is outside the nucleus are higher than the corresponding doses shown in Fig. 1. However, when the initial location of the sources is in the nucleus we don’t observe such a significant increase in dose to the nucleus relative to when the initial sources locations are outside the nucleus, for all four radionuclides. This data was generated using the g4em-dna physics in the nucleus and condensed history electromagnetic physics in the remainder of the cell geometry. This is due to an issue with the modelling of the ionisations caused by the heavy daughter nuclei from the radioactive decay. This current work was performed with OpenTOPAS and TOPAS-nBIO that is built using Geant4 v11.1. In this version of Geant4, when using g4em-dna physics, ionisation physics is only modelled for a limited number of light ions, not the heavier daughter ions generated by the radioactive decay of the four parent radionuclides used in this current study (*Geant4DNA—Physics Processes and Models—Ions*, n.d.). The radioactive decay of the parent is modelled by the G4RadioactiveDecay physics generating decay products including the heavy daughter ions with their small recoil energy. However, when the initial location of the primary radionuclide sources is in the nucleus the heavy daughter ions are transported out from the nucleus without any ionisation interactions and are unlikely to undergo alpha decay in the step to the nucleus boundary. Dose deposition in the nucleus due to beta and alpha particles from the initial parent decay will be modelled however so some dose will still be scored in the nucleus, just not as high as expected if all the daughters (and granddaughter) interactions were modelled. For initial source locations outside the nucleus (in the cell wall, cytoplasm and on the nucleus wall) condensed history electromagnetic physics lists do model the interactions of heavy daughter ions. Interestingly, Geant4 v11.2 includes a scaling method to include ionisations of heavier ions so future versions of TOPAS-nBIO based on more recent Geant4 version should at least have some capacity to model the ionisation interactions of heavy ions if not as accurately as one would like.

With this limitation in mind we performed simulations with the sources modelled as isotropic pure alpha emitters with discrete energies and relative spectral weights given by the decay schema in Appendix 1. Sources were randomly located in the different initial locations as previously. These results are shown in Fig. 3. In this case we do see the increase in dose to the nucleus/decay when the sources are initially located in the nucleus, relative to when the sources are initially outside the nucleus geometry. The variation in dose to the nucleus/decay does not change very much with radionuclide when they are modelled as purely alpha-emitters. This indicates the alpha energy is not that important in the efficacy of the dose delivery to the nucleus with only one alpha/decay for this source model. Comparing the data in Figs. 2 and 3, where the full decay process was modeled showed how radionuclides with more complex decay schema such as ^225^Ac and ^223^Ra are able to deliver more dose to the nucleus/decay.

The data discussed thus far have only considered the absorbed dose to the nucleus/decay and the number of particles crossing the nucleus wall. While at the macroscopic level absorbed dose is considered a surrogate for DNA damage, the real target for therapeutic radiation interventions is the irreparable damage to nuclear molecular DNA. This can be quantified in terms of the double strand break (DSB). Figure 4 shows the results of simulations where the DBSCAN algorithm was used to determine an approximation to the number of single strand breaks and double strand breaks (both simple and complex, sDSB and cDSB respectively). Full decay was modelled for the different sources and g4em-dna physics used in the nucleus. Similar trends in the SSB, sDSB, and cDSB/decay are seen as for the absorbed dose to the nucleus/decay. As previously discussed, the same caveat regarding the data when the sources are initially in the nucleus applies here, i.e. the g4em-dna physics is not modelling the ionisation interactions of the heavy daughter ions and so they leave the nucleus without contributing any SSB, sDSB, and cDSB. With this in mind we also applied the DBSCAN algorithm to scoring SSB, sDSB, and cDSB when discrete energy alpha sources were used. g4em-dna physics was used in the nucleus. The results are shown in Fig. 5. The data confirms the idea that for targeted alpha therapy maximum efficacy can be achieved when the alpha emitting sources are delivered into the cell nucleus or at the very least able to attach to the nucleus wall. The data in Fig. 5 (g,h,i) also shows the proportion of strand breaks due to alpha’s is also higher when the sources are initially in the nucleus.

Table 2 compares this current work with that of Guerra Liberal et al. [14], also performed using TOPAS for ^211^At. They used an alpha source model with condensed history physics. Best agreement is found for the alpha contribution to the dose to the nucleus/decay with a full decay model using the condensed history electromagnetic physics. Key observations from the data in Table 2. Use of g4em-dna physics in the nucleus results in an increase in the dose to the nucleus/decay regardless of the initial source location noting the issues with heavy daughter ion ionisation modelling when sources are initially in the nucleus. Modelling the full decay of ^211^At shows an increase in the dose to the nucleus/decay compared to if only alpha’s are modelled or the alpha dose only is scored. This indicates that it is not only the direct alpha ionisations that are important for the efficacy of targeted alpha therapy. The low energy electron spectra (< 100 keV) for each of the radionuclides is shown in Fig. 6. The discrete peaks in these spectra can be attributed to two processes (1) Internal conversion electrons emitted with energies equal to the nuclear transition energies minus the discrete binding energies of the atomic electrons in the daughter nucleus and (2) Auger electrons emitted with energies characteristic of the daughter atoms resulting from the filling of inner-shell vacancies created during electron capture and internal conversion processes. These energies are determined by the discrete binding energies of the electron shells involved in the Auger transitions. From the decay schema of ^211^At in appendix 1 we see 58% of parent decays are through electron capture. This will result in the emission of discrete low energy auger electrons and as previously discussed these are known to be densely ionising. These discrete low energy electrons are of significant radiobiological importance, as clustered DNA damage is generally considered more cytotoxic than isolated damage [24].

While the insights obtained from this study are valuable, their accuracy can be enhanced by incorporating additional parameters into the monte-carlo simulations, such as realistic cell geometries, complete chemical interactions, mitochondrial DNA damage, and cell cycle determinations. Furthermore, using more authentic DNA models using TOPAS-nBio would increase accuracy beyond that achieved through the DBSCAN algorithm.

Another consideration regarding α emitting radiopharmaceuticals is the distinct chemical behaviour of α emitting daughter radionuclides, produced through the decay of primary α radionuclides. These daughter radionuclides exhibit varying chemical properties which can lead to unstable bonds with carrier molecules. Consequently, they may quickly dissociate from bifunctional chelates conjugated to carrier molecules. When the recoil energy surpasses ∼ 100 keV, it exceeds the binding energy between the radionuclide and the targeting molecule. As a result, these liberated α emitting radionuclides can irradiate non-target organs, potentially causing long term complications. This concern is particularly relevant for radionuclides that decay by emitting multiple α particles, such as ^225^Ac and ^223^Ra. One strategy to overcome this problem involves a novel approach in the preparation of α-emitting radiopharmaceuticals. This strategy entails incorporating radionuclides into nano-carrier systems such as liposomes or nanoparticles. These bio-nanoconstructs can deliver a targeted dose and are swiftly cleared from the body after targeting, minimising radiation exposure to non-target organs.

## Conclusions

Monte-carlo simulations conducted using the TOPAS-nBio extension of the TOPAS monte-carlo code, using Geant4-DNA models, have been shown to be a valuable tool for estimating absorbed dose at cellular and subcellular levels. Although there has been growing interest in modelling TαT, previous research has predominantly focused on β-emitters, this study highlights the versatility of TOPAS-nBio in microdosimetry and DNA damage quantification involving α emitting radionuclides. With the development of innovative radiopharmaceuticals, these findings offer insights into identifying the most effective cellular targets to enhance cytotoxicity within cancer cell nuclei. Additionally, it was demonstrated that when subcellular dosimetric assessments are paired with the quantification of DNA damage resulting from emissions during the decay of α emitting radionuclides, it facilitates the determination of DSB to S-value and DSB to SSB ratios. This clarifies which potential radionuclides exhibit high relative efficacy regarding ionisation and energy deposition characteristics for optimal therapeutic impact. The study has also highlighted the caution that should be employed when modelling radioactive decay of heavy radionuclides with the g4em-dna track structure physics models. We look forward to future versions of topas-nBio being built on more recent Geant versions that should help improve the accuracy of modelling the ionisation physics of the heavy daughter ions.

## Appendix

Ac-225 decay chain

Ra 223 Decay chain

Pb212 Decay chain

At-211 Decay chain
